# Partial Replacement of Soybean Meal with Canola Meal or Corn DDGS in Low-Protein Diets Supplemented with Crystalline Amino Acids—Effect on Growth Performance, Whole-Body Composition, and Litter Characteristics

**DOI:** 10.3390/ani12192662

**Published:** 2022-10-04

**Authors:** Adeleye M. Ajao, Dima White, Woo K. Kim, Oluyinka A. Olukosi

**Affiliations:** Department of Poultry Science, University of Georgia, Athens, GA 30602, USA

**Keywords:** low-protein, amino acids, canola meal, corn DDGS, broiler chickens

## Abstract

**Simple Summary:**

Dietary protein reduction with amino acid supplementation is a nutritional approach to reducing environmental pollution. Soybean meal is nutritionally superior to alternative protein feedstuffs, such as corn distillers dried grain with solubles (cDDGS) and canola meal (CM), because of its relatively good balance of amino acids. This study investigated the usage of CM or cDDGS compared to SBM in low-protein broiler diets and their effects on growth performance, carcass yield, whole-body composition, and litter characteristics. The results demonstrated that replacing soybean meal with cDDGS or CM negatively affected the performance of the birds, whereas reducing dietary protein reduced the litter surface ammonia, irrespective of protein feedstuff. Therefore, complete replacement of SBM with cDDGS or CM in low-protein diets is not feasible for optimum performance in broiler chickens.

**Abstract:**

A 42-day study was conducted to explore the application of supplemental amino acids (AA) in low-protein diets with soybean meal (SBM), canola meal (CM) or corn distillers dried grain with solubles (cDDGS) as the main protein feedstuffs. The responses of interest were growth performance, carcass yield, whole-body composition, litter ammonia and litter N. On d 0, a total of 540 Cobb 500 (off-sex) male broilers were allocated to 36 floor pens. All the birds received one starter diet that met nutrient requirements during the first 10d. Thereafter, six experimental diets were provided in grower and finisher phases. The diets included a positive control (PC): a corn–SBM diet with adequate protein. The protein level of the negative control (NC) was decreased by 45 g/kg relative to the PC. The next two diets had the same protein levels as the NC but with cDDGS added at 50 or 125 g/kg. The last two diets had the same CP as the NC but with CM added at 50 or 100 g/kg. All the low-protein diets had the same level of standardized ileal digestible indispensable AA according to Cobb 500 recommended level. Gly and Ser were added as sources of non-specific N. The dietary protein reduction in corn–SBM diets at both phases decreased (*p* < 0.05) weight gain and increased (*p* < 0.05) feed conversion ratio (FCR). Increasing levels of cDDGS or CM, at a constant CP level, linearly decreased (*p* < 0.05) the weight gain and feed intake, whereas increasing CM level linearly increased (*p* < 0.05) FCR in the grower and finisher phases. The eviscerated and carcass yields decreased, whereas the fat yield increased (*p* < 0.05) with reduced protein in corn–SBM diet. Increasing levels of cDDGS and CM at a constant CP level quadratically decreased (*p* < 0.05) the eviscerated weight, whereas the fat weight linearly decreased (*p* < 0.05) with increasing levels of cDDGS and CM. The birds receiving the PC diet had a lower (*p* < 0.05) lean muscle (%) and a higher fat (%) compared to birds receiving the NC diet at d 21. However, on d42, birds receiving the PC diet had decreased (*p* < 0.05) bone mineral density, bone mineral content and lean weight compared to those receiving the NC diet. The litter ammonia increased (*p* < 0.05) with the increasing levels of protein in the SBM diets. In conclusion, 50 g/kg inclusion levels of CM and cDDGS at the same low-protein levels as SBM produced a similar growth response to the NC, whereas higher levels were detrimental. Hence under the conditions of the current experiment, complete replacement of SBM with DDGS or CM in low-protein diets was not feasible.

## 1. Introduction

The increase in the world population brings about a spike in the demand for poultry meat. This demand is driven mainly because of poultry products’ accessibility, affordability and overall acceptance across a variety of traditions, cultures and religions [1]. However, to meet this increased demand and to sustain a high rate of growth of broilers, high-protein diets are fed to birds. This poses environmental (e.g., release of gases, such as NH_3_ and N) and bird welfare concerns [2]. In addition, governmental policies and environmental agencies have increased pressure on producers to lower NH_3_ emissions [3,4].

One leading approach to mitigating excess N excretion is a reduction in dietary protein and supplementing such diets with limiting amino acids (AA). Several broiler experiments have demonstrated that supplementing low-protein diets with appropriate supplemental AA resulted in a growth performance equal to that of an adequate-protein diet but with a reduction in litter N [5,6] and/or ammonia [7]. However, nearly all studies on reduced protein diets with supplemental AA have used SBM as protein feedstuff. This is likely because SBM has a high protein content and a nutritionally superior AA balance [8,9]. Roberts et al. [10] reported that ammonia emissions can be diminished when feeding birds a fibrous diet because: (1) AA in a highly fibrous diet is less digestible compared to that in a low fibrous diet; and (2) AA in a fibrous diet is less likely to degrade to the urea and consequently leads to NH_3_, because dietary fiber increases the metabolism and growth of beneficial bacterial in the large intestine [11]. 

Canola meal (CM) and corn distillers dried grain with solubles (cDDGS) are potential alternative protein feedstuffs currently used in poultry diets in many parts of the world. As with many less-conventional feedstuffs, the chemical composition of the feedstuffs is variable but they are sufficiently high in CP and AA, which make them attractive protein feedstuffs in poultry diets [12]. Roberts et al. [10] reported that the use of cDDGS and CM (which increased dietary fiber) lowered NH_3_ emissions from manure over 7 days in comparison to the positive control. The use of cDDGS in low-protein broiler diets has not been studied extensively. For example, Guney et al. [13] showed that up to 200 g/kg of low-fiber cDDGS could be added to broiler starter diets without any detrimental effects on live performance.

Previous research has demonstrated that older birds could tolerate up to 150 g/kg of cDDGS in broiler diets [14,15,16,17]. Zhu et al. [18] observed that FCR increased with increasing CM level (0 to 294 g/kg) in layers’ diets.

To the authors’ knowledge, although there is abundant information on the use of SBM in low-protein diets, information on the use of CM or cDDGS in low-protein diets is currently unavailable. Therefore, the objective of the current experiment was to investigate the use of increasing levels of CM or cDDGS compared to SBM in low-protein broiler diets. A diet adequate in protein was included to ascertain the influence of reduced protein in SBM diets. The response criteria of interest were growth performance, carcass yield, whole-body composition and litter characteristics. 

## 2. Materials and Methods

All animal experiment procedures used in the current study were approved by the Institutional Animal Care and Use Committee of the University of Georgia.

### 2.1. Animals and Diets Experimental Design 

A 42-day experiment was conducted to investigate the effect of supplemental AA in low-protein diets with SBM or diets in which graded levels of CM or cDDGS partly (or nearly totally) replaced SBM. Five hundred and forty Cobb 500 (off-sex) male broiler chicks were obtained on the day of hatch (d 0) and allocated on the basis of body weight into 36 floor pens in an environmentally controlled room. All the birds received the same corn–SBM starter diet during the first 10 d, and experimental diets were fed from d 10 to 42.

The six experimental diets included positive (PC) and negative control (NC) diets formulated to be adequate or protein-deficient, respectively. The PC and the NC diets in grower phase (d 10–28), had 185 vs. 140 g/kg CP, respectively. For the finisher phase (d 28–42), the PC and the NC diets had 170 vs. 130 g/kg protein levels, respectively. The remaining four diets had the same protein level as NC. SBM was replaced with 50 or 125 g/kg cDDGS (cDDGS50 and cDDGS125, respectively) or with 50 or 100 g/kg CM (CM50 or CM100, respectively). All the six diets were formulated to be similar in digestible essential AA according to Cobb 500 requirement [19]. All the low-protein diets had supplemental Gly and Ser as sources of non-specific N.

The model used in the current study was of diets in which nonphytate P and Ca levels were reduced and subsequently supplemented with phytase. Phytase (Quantum Blue, AB Vista) was supplemented at the rate of 0.1 g/kg to provide 500 phytase units per kg diet (one phytase unit is the activity required to release 1 µmoL of inorganic P per minute at pH 5.5 from an excess of 15M sodium phytate at 37 °C). The matrix values used for Ca and nonphytate phosphorus were 1.75 and 1.5 g/kg, respectively, according to manufacturer’s specification. Because there were no specific requirements for Gly and Ser, Gly-equivalent value used in the low-CP diets was based on the Gly-equivalent level of the PC diet. The diets were fed as pellets throughout the experiment (as crumbs on d 0 to 10).

Analyzed values of feedstuffs (SBM, CM and cDDGS) used are shown in Table 1. Diet formulas and calculated nutrient values are shown in Table 2, Table 3 and Table 4. Feed and water were provided ad libitum during the study. Temperature and light schedules followed Cobb 500 management guide.

### 2.2. Growth Performance, Whole-Body Composition, Litter Surface Ammonia Measurement and Carcass Composition

Birds and feed were weighed on d 0, 10, 28 and 42. Mortality was monitored daily and used to calculate mortality-adjusted weight gain (WG), feed intake (FI) and FCR. On d 21 and 42, one bird from each pen was randomly selected for whole-body composition analysis using dual-energy X-ray absorptiometry (DEXA; pDEXA®, Bone Densitometer, Norland Medical System Inc., Fort Atkinson WI, USA). The whole bird was defined as a region of interest. Whole body region scans were conducted to measure bone mineral density (BMD), bone mineral content (BMC), total area of bone, total weights of lean and fat, as well as lean and fat percentages. Scanning was performed across each bird, placing each bird at the same position and orientation during the measurement. All scans were obtained at a scan speed of 2.5 mm/s, with a voxel resolution of 0.07 × 0.07 × 0.50 mm. 

Total ammonia produced at the litter surface was determined for each pen on d 42. Ammonia concentrations were determined using a Drãger® chip measurement system (CMS portable gas meter, Drägerwerk AG & Co. KGaA Moislinger Allee 53–55 23558 Lübeck, Germany). Ammonia was measured by placing 0.03 m^3^ plastic box on the bedding in an area to the side of the feeder where no caked litter was present. A tube connected the box with the Drager® CMS. The box was left on the bedding for 2 min with the Drãger ® CMS pump running. Ammonia concentration (ppm) was measured after 2 min. Litter samples close to the water lines were collected on d 42 for litter N determination. 

Two birds were randomly selected from each pen on d 42, after feed was withdrawn overnight (but with access to water) and slaughtered for carcass yield evaluation. The following data were collected from the carcasses: eviscerated carcass yield, breast yield, wings yield, thighs and drumsticks, and back plus ribs. Carcass yield was calculated as:

$$\mathrm{Eviscerated}\;\mathrm{Carcass}\;\mathrm{Yield},\;\%\;=\frac{Eviscerated\;carcass\;weight,\;\;g}{Live\;weight,g}\times 100;\;$$

whereas cuts (e.g., breast) yield was calculated as:

$$\mathrm{Breast}\;\mathrm{yield},\;\%=\frac{Breast\;weight,\;g}{Eviscerated\;Carcass\;weight,\;g}\times 100.$$

### 2.3. Chemical Analysis

All the diets and feedstuffs were analyzed for chemical profiles using AOAC [20] procedures. Samples were dried at 100 °C for 24 h to determine the dry matter (method 934.01). Nitrogen content was measured using the combustion method (method 968.06). Amino acids were analyzed AOAC procedures (Method 994.12). Briefly, the samples were hydrolyzed with 6 N HCl containing phenol for 24 h at 110 ± 2 °C in glass tubes in an oven. Amino acids were measured using AA analyzer (ion exchange) with ninhydrin post-column derivatization. The chromatograms detected at 570 and 440 nm were integrated using dedicated software (Agilent Open Lab software, Waldbronn, Baden-Württemberg, Germany). Cys and Met were analyzed as cysteic acid and methionine sulphone, respectively, by oxidation with performic acid–phenol for 16 h at 0 °C prior to hydrolysis. For the measurement of Trp, the samples were saponified under alkaline conditions with barium hydroxide solution in the absence of air at 110 °C for 20 h in an autoclave. The internal standard α-methyl Trp was added to the mixture following hydrolysis. After adjusting the hydrolysate to pH 3.0 and diluting with 30% methanol, Trp and the internal standard were separated by reverse phase chromatography (RP-18) on an HPLC column (CORTECS C18 Column; 2.7 µm, Waters Corporation, Dublin, Ireland). Finally, detection was selectively performed by means of a fluorescence detector to prevent interference by other AAs and constituents.

### 2.4. Statistical Analysis

Data were analyzed as appropriate for randomized complete block design using the MIXED procedure of SAS 9.4. The blocks were treated as random variables whereas treatments were the fixed effects. Although all the birds received the same diet until d 10, in order to account for inevitable variation in body weight, d 10 body weight (252 ± 11 g) was used as covariate in the statistical analysis. Orthogonal polynomial contrasts were used to determine the linear and quadratic responses to increasing levels of CM or cDDGS in low-protein diets. When both linear and quadratic responses are significant, only the quadratic response is discussed. One df pair-wise contrast was used to determine the effect of protein reduction between the PC and the NC diets. Statistical significance was set at *p* ≤ 0.05. Data can be found in Supplementary Materials Table S1.

## 3. Results

The analyzed CP and AA contents of the experimental diets (starter, grower and finisher) were close to the expected values (Table 5 and Table 6). The analyzed AA content was the total AA, but the values reflected the expected variability due to the inclusion of supplemental AA in the applicable diets. The average analyzed phytase activity was 1278 FTU/kg, which was far in excess of the expected level of 500 FTU/kg.

### 3.1. Growth Performance

Birds on the PC diet were heavier and had a lower (*p* < 0.05) FCR compared to those on the NC diet in the grower (d 10–28) and finisher (d 28–42) phases (Table 7). In diets with similar levels of protein and digestible AA, increasing the dietary levels of cDDGS at the expense of SBM in the low-protein diets linearly reduced (*p* < 0.05) weight gain and feed intake in the grower phase but had no effect in the finisher phase. During the grower and finisher phases, increasing the dietary level of CM at the expense of SBM linearly increased (*p* < 0.01) the FCR but had no effect on feed intake. In addition, increasing the CM level in low-protein diets linearly reduced (*p* < 0.05) the weight gain during the finisher phase only. The day 42 BW was lower in the NC compared to the PC (*p* < 0.05). Increasing levels of cDDGS or CM in the low-protein diets linearly reduced (*p* < 0.05) the d 42 BW.

### 3.2. Carcass Yield 

Birds receiving the NC diet had a lower (*p* < 0.05) eviscerated weight and carcass yield, but a greater (*p* < 0.05) fat yield and thigh yield compared to those receiving the PC diet (Table 8). Increasing the cDDGS level in the low-protein diets quadratically decreased (*p* < 0.05) the eviscerated weight, abdominal fat weight and fat yield (%), as well as linearly increased (*p* < 0.05) the wing yield but had no effect on the carcass yield, breast meat yield, thighs yield or back and ribs yield. Increasing the CM level in low-protein diets quadratically decreased (*p* < 0.05) the eviscerated weight, as well as linearly reduced (*p* < 0.05) the abdominal fat weight and fat yield (%); but had no effect on other responses. 

### 3.3. Whole Body Composition

On d 21, birds receiving the NC diet had a lower (*p* < 0.05) lean muscle (%) and a higher fat (%) compared to the birds receiving the PC diet (Table 9). Increasing the levels of cDDGS in the low-CP diet, at the expense of SBM, linearly decreased (*p* < 0.05) the bone mineral density, bone mineral content and total area of bone as well as the total weight of fat, lean muscle and fat (%) at day 21 (Table 9). 

On d 42, birds receiving the NC diet had lower (*p* < 0.05) bone mineral density, bone mineral content and lean weight compared to those receiving the PC diet (Table 10). However, increasing the cDDGS or CM levels in the low-protein diet, at the expense of SBM, had no significant effect on any of the responses on d 42.

### 3.4. Litter Surface Ammonia and Litter Nitrogen

Ammonia was not detectable in the litter surface when measured on d 0. The litter NH_3_ was lower in birds receiving the NC diet (*p* < 0.05) compared to those receiving the PC diet. The inclusion of cDDGS or CM in the low-protein diet, at the expense of SBM, had no effect on the litter NH_3_ on d 42, irrespective of the protein feedstuffs used (Table 11). The litter N and litter N per kg body weight gain were not significantly affected by the treatments.

## 4. Discussion

The objective of the current study was to assess the growth performance response, carcass yield, whole-body composition, litter ammonia and N levels for broilers fed low-protein diets with supplemental AA using SBM, cDDGS or CM. Due to the fact that many studies investigating low-protein diets have used diets that are limiting only in protein or AA, in the current study, we utilized diets corrected for Ca and non-phytate P reduction with phytase supplementation in order to mimic standard practice with phytase supplementation. Because all the diets had the same level of phytase, Ca and non-phytate P, the responses obtained can be attributed solely to treatment differences in the level and type of protein feedstuff used. In addition, in order to partition the growth performance responses to phase-specific effects, the body weight at the end of starter phase (d10) was used as a covariate in the analysis of growth performance in both the grower and finisher phases. 

Soybean meal is well suited to be the plant protein feedstuff of choice in low-protein AA-supplemented diets. The promotion of alternative protein feedstuffs, such as CM and cDDGS, in low-protein diets compared to SBM is popular because these feedstuffs have a relatively well-balanced amino acid profile [21]. 

The growth performance of broiler chickens receiving the NC diet in the current study was lower than those receiving the PC diet both in the grower and finisher phases, even though both diets were supplemented with essential AA, including glycine-equivalent to meet the Cobb 500 requirement. This is similar to the observations of several other studies [22,23,24]. However, other authors have reported a comparable performance for broiler chickens fed adequate or low-protein diets [5,6,7]. The poorer performance of birds receiving the NC diet in the current study could be partly explained by the drastic reduction in dietary protein (185 vs. 140 g/kg for grower diet) as a consequence of the drop in SBM inclusion from 265 to 75 g/kg in the PC vs. the NC diets, respectively. This 72% reduction in the dietary SBM level implied a reduction in intact protein in the low-protein diets. On one hand, reducing the amount of protein reaching the hindgut can be beneficial because it translates to less substrates from which putrefactive bacteria in the gastrointestinal tract would generate harmful substances, such as amines or phenols, that may impair growth [25,26,27]. Nevertheless, a comparatively poorer performance can result from such diets due to the inherently different digestion location and dynamics of ‘intact’ protein and non-bound amino acids. The implications may include an insufficient N pool for the synthesis of non-essential amino acids, dietary imbalances between assumed essential and non-essential amino acids and the possibility that the amino acid requirements have not been identified with sufficient accuracy in the context of reduced-protein diets [28,29,30]. In addition, the lower feed intake in the NC diet (more markedly different in the finisher phase) may account for the reduced weight gain. Protein, and consequently SBM, reduction in the NC diet of the current study was accompanied by the addition of corn starch and cellulose. These dietary modifications will result in increased dietary starch:protein ratio, with consequences on foregut digestion and distal digestive tract fermentation pattern and implications on the weight gain response [31].

By far, the majority of studies utilizing low-protein diets have focused on SBM [32,33,34,35]. However, other plant protein feedstuffs (such as cDDGS and CM) are widely used in broiler diets in many parts of the world. Consequently, one main aim in the current study was to compare the effect of the partial or near complete replacement of SBM with cDDGS or CM while maintaining the same level of standardized digestible AA. Corn DDGS is characterized by a comparatively high protein content and has been studied in many broiler experiments [12,15,36]. However, these studies have incorporated cDDGS for broilers in diets with adequate protein, not in the context of low-protein diets as used in the current study. 

In the current study, birds receiving 125 g/kg of cDDGS in the grower phase had a markedly lower weight gain and feed intake compared with the corn–SBM diet at the same protein level. The high cDDGS inclusion level resulted in near total replacement of SBM and higher supplementation levels of supplemental AA. However, no significant weight differences as a result of the cDDGS addition to the diets were observed during the finisher phase. This may indicate that the weight gain effect of the cDDGS replacement of SBM is age- or phase-dependent, or that an adaptation of the birds to the diet had taken place [37,38]. In studies utilizing high dietary levels of cDDGS (although not in low-protein diets), Campasino et al. [39] reported a lower body weight gain in broiler chickens fed 150 g/kg cDDGS compared to those fed diets containing 0 or 50 g/kg DDGS. The highest cDDGS level used in the current study is 25 g/kg lower than Campasino et al. [39]; the effect of high cDDGS inclusion may have been exacerbated by low dietary protein.

The birds receiving 100 g/kg CM had a lower weight gain during the finisher phase, and a higher FCR at both the grower and finisher phases, compared to the broilers receiving SBM. Previous studies [40,41] also reported a decrease in performance when more than 150 g/kg CM was added in the diet of broiler chickens. These observations could be attributed to the high starch content relative to protein in CM, which in turn changes the digestive dynamics in the birds as a result of increasing the dietary starch:protein ratio, leading to poor digestibility as a result of the negative impact on the intestinal structure and function [21,31]. Liu et al. [42] observed that starch is more rapidly taken up than protein in the intestine of broilers fed a sorghum-based diet. Selle et al. [43] suggested that the important role that starch plays in the intestinal passage rate and digestive dynamics is the reason for its rapid digestibility when compared to protein in the gut. Moss et al. [44] observed that birds fed low protein diets with a higher starch content will flood their intestine with glucose which will compete with amino acids for absorption through their shared sodium dependent pathways, thereby causing poor digestibility. In addition, Mc Neill et al. [45] attributed the poorer performance in CM to the presence of a trypsin inhibitor, which would have consequences on the feed intake as well as nutrient utilization.

The eviscerated weight and carcass yield in the current study were lower for birds receiving the NC compared to the PC diet. Others have similarly observed that the carcass yield becomes inferior in broilers fed low-protein diets with more than a 30 g/kg protein reduction, even when all known nutrient requirements are met [22,28,46]. Others have observed that dietary protein reduction has no effect on the carcass yield of broiler chickens even when the reduction is 30 g/kg, or higher [7,47]. It is reasonable to expect that lower live body weight as a consequence of the dietary protein reduction will produce a lower eviscerated carcass weight unless the nutritional intervention produced a differential accretion of the economic and non-economic parts of the carcass. The result of the current experiment suggests otherwise, in that the carcass yield responses followed the same pattern observed for the weight gain responses to both reduced protein as well as the replacement of SBM with cDDGS or CM. 

In the current study, broilers fed low-CP diets accreted 10% more abdominal fat compared to those fed an adequate protein diet. It is known that the protein and AA levels of the diets influence the carcass composition of broiler chickens, and that decrease in dietary protein usually precipitates a decrease in the carcass protein and an increase in the carcass fat content [48]. The explanation for the increase in abdominal fat with reduced protein has been attributed to the increased ME:protein ratio leading to poor digestibility as a result of the negative impact on the intestinal structure and function [49,50]. Because all the diets in the current study were isocaloric, in the content of reducing the protein level, the increased ME:protein ratio is an inevitable consequence of the nutritional modification.

The abdominal fat weight and fat yield were lower in birds receiving cDDGS and CM compared to those receiving the NC diet, reflecting the disparities in body weight. This could be attributed to the presence of relatively high fiber content in cDDGS when compared to SBM, thereby leading to lower nutrient digestibility as a result of the negative impact on the intestinal structure and function [37,38,40,51]. On the other hand, the abdominal fat weight and fat yield were greater (or comparable to the NC diet) in diets with lower inclusion levels of cDDGS and CM. Similar observation have been made in CM diets [40,51]. In the case of CM, it has been suggested that the presence of high polyunsaturated fatty acid in CM compared to SBM may decrease the fat deposit in animals [52].

Although there were treatment effects on the carcass and fat yields, there were only marginal treatment effects on the yields of breast meat, thigh, and back and ribs. This is similar to the observations of Kobayashi et al. [53] and suggests that even though dietary protein level modification may influence weight gain or abdominal fat accretion, its influence on carcass cuts is less pronounced. The effects of reduced protein on growth performance may not be mirrored by the effect on carcass cuts. Possible explanations are the possible differences in the effect of dietary modifications on total weight gain or the growth of specific parts and organs. For example, the effect of dietary manipulation may be manifested to a greater degree in the growth and development of the digestive organs but not the breast muscle. Under such circumstances, the effect is seen in the total weight of the animal but not in the carcass cuts. This is why, depending on objective of the experiment, it is important to complement data on weight gain with those on carcass cuts; the latter representing the parts of the birds that are of greater commercial importance [54,55].

A DEXA scan provides an accurate, in-depth body composition analysis of a bird for its fat, muscle, bone and water components. This analysis is relevant, as it can be used to identify health risks and other problems, such as metabolic bone disorder, before they occur [56,57]. In the current study, the scan on d 21 showed that the whole-body fat (%) increased in the NC compared to PC, whereas the lean muscle (%) followed the opposite pattern. This could be attributed to the increased energy to protein ratio in the low CP diets. However, bone characteristics were not affected by the reduction in dietary protein. There were no differences among the treatments during the first 3 weeks of age; this observation could be explained by the proposition that their early life is a period when the birds channel most of their nutrients into organ development and overall intestinal growth [58,59,60].

On the other hand, BMC, BMD, total bone area, fat weight, lean weight, lean muscle % and fat % linearly decreased with the increasing level of cDDGS (lean muscle % increased linearly with the increasing level of cDDGS). The decrease in these responses was more pronounced in the cDDGS50 diet (compared to the NC), and the further addition of cDDGS in the diet did not produce further significant responses. These responses might be attributed to limited ability of broiler chickens in the grower phase (d 10 to 28 in the current study) to utilize a relatively more fibrous cDDGS diet [37,38]. 

For the scans performed on d 42 in the current study, the only significant observations were of the effect of protein reduction on BMD, BMC and lean weight. Bone mineral density (BMD) is important for diagnosing osteoporosis [61,62]. In broilers, bone strength is relevant both for carcass quality and bird welfare [63]. By d 42 in the current study, both BMD and BMC were lower in all the birds receiving low-protein diets compared to the PC diets. Yang et al. [64] observed no significant effects of different protein dilution levels of broiler starter diets on bone responses. A likely explanation for the observation in the current study regarding the effect of protein reduction is the overall reduction in the feed intake of birds receiving the low-protein diet. The implication of this is a reduction in digestible nutrient intake, including minerals. The reduced mineral intake may be responsible for a reduced bone mineralization, which is observable as bone mineral density. 

The lean tissue weight from the DEXA scanning is synonymous with the muscle weight [65]. Therefore, it is no surprise that result observed in lean tissue weight at d 42 in the current experiment followed a similar trend to the carcass weight of broilers at d 42. This result is similar to that of others [66,67], who have observed that feeding broiler chickens low-protein diets impairs their lean muscle weight.

The litter NH_3_ in the current study decreased with the 45 g/kg CP reduction. Our observations of the litter surface ammonia response to the protein reduction in the current experiment are similar to those of others [68,69], who have reported reduced litter NH_3_ in birds receiving low-CP diets, which can be explained by the reduced water intake in birds receiving low-protein diets due to a decreased need for water to excrete excess N [70]. Others [69,71] also reported significant reductions in ammonia emissions following relatively modest reductions in dietary protein levels. However, litter N (%) and litter N per kg of body weight were not affected by the dietary CP reduction in the current study. The implication, therefore, is that the dietary protein reduction, per se, and not necessarily the feedstuff, is the primary factor for the reduced litter N and NH_3_.

## 5. Conclusions

In conclusion, under the conditions of the current experiment, reducing dietary protein by 45 g/kg in corn–SBM diets produced an inferior growth performance, carcass yield and altered whole-body composition (on d 21) but also beneficially reduced the litter surface ammonia. In addition, the partial or near-total replacement of SBM with cDDGS or CM in the low-protein diets had a further negative effect on growth performance. Therefore, complete replacement of SBM with cDDGS or CM in low-protein diets as used in the current experiment is not feasible.

## Figures and Tables

**Table 1:** Chemical composition (g/kg, as-fed basis) of protein feedstuffs used in experimental diets.

	Soybean Meal	Canola Meal	Corn DDGS
Crude protein	491	319	381
Essential AA			
Arg	33.5	22.4	13.4
His	12.3	9.8	8.1
Ile	23.1	15.8	11.6
Leu	36.4	26.4	33.1
Lys	29.7	21.3	10.0
Met	6.3	6.6	6.2
Phe	24.5	16.3	14.3
Thr	18.1	15.1	11.2
Trp	6.4	4.2	2.2
Val	23.7	19.2	14.9
Nonessential AA			
Ala	20.5	16.0	20.2
Asp	52.8	28.4	18.6
Cys	6.7	8.5	6.2
Glu	86.2	63.6	41.7
Gly	20.1	17.9	11.4
Pro	22.7	20.1	22.3
Ser	21.0	14.1	12.7
Tyr	16.6	10.7	10.6

**Table 2:** Ingredient and chemical compositions (g/kg) of starter diets (day 0 to 10).

Items	Starter (d 0–10)
Corn	527.4
Soybean meal	363.0
Soybean oil	44.0
Corn starch	30.0
Dicalcium phosphate	10.0
Limestone	9.0
L-Lysine·HCl	0.20
DL-Methionine	1.20
L-Threonine	0.30
Sodium bicarbonate	2.0
Salt	2.80
Vitamin premix ^1^	5.0
Trace minerals premix ^2^	5.0
Phytase ^3^	0.10
Total	1000
Calculated nutrients (g/kg) and energy	
Protein	220
ME, kcal/kg	3011
Ca	7.23
P	5.49
Available P ^3^	2.98
Digestible amino acids, g/kg	
Arg	14.9
His	5.9
Ile	9.6
Leu	18.7
Lys	12.3
Met	4.6
Cys	3.6
Phe	10.9
Tyr	8.0
Thr	8.7
Trp	2.6
Val	10.5
Gly	7.1
Ser	9.2
Gly-equivalent	13.7
Phe+Tyr	18.9

^1^ Vitamin A, 3,527,360 IU/kg; vitamin D3, 1,399,921 ICU/kg; vitamin E, 19,400 IU/kg; vitamin B12, 8.8 mg/kg; menadione, 1102 mg/kg; riboflavin, 3527 mg/kg; d-pantothenic acid, 5467 mg/kg; thiamine, 970 mg/kg; niacin, 20,282 mg/kg; vitamin B6. 1455 mg/kg; folic acid, 573 mg/kg; biotin, 79 mg/kg. µg. ^2^ Ca, 3.20–4.20%; Mn 13.40%; Zn, 10.70%; Mg, 2.68%; Fe, 2.63%; Cu, 40,000 ppm; I, 1000 ppm; Se, 400 ppm. ^3^ Available P level excluded the matrix for the phytase (Quantum blue). Gly-equivalent = Gly + (Ser × 0.714).

**Table 3:** Feedstuffs and chemical compositions (g/kg) of grower phase diets (d 10 to 28) fed to broilers receiving diets containing soybean meal with reduction in crude protein or low-protein diets with serial inclusion levels of canola meal or corn–DDGS supplemented with crystalline amino acids.

Items	PC	NC	cDDGS50	cDDGS125	CM50	CM100
Corn	651	683	686	572	675	570
Corn–DDGS			50.0	125		
Canola meal					50.0	100
Soybean meal	265	75.0	36.0	3.00	30.0	7.00
Soybean oil	46.0	32.0	28.0	20.0	36.0	40.0
Cellulose filler		54.0	52.0	60.0	61.0	76.0
Corn starch		72.0	60.0	128	62.0	120
Dicalcium phosphate	8.00	10.1	9.40	8.40	10.0	10.1
CaCO_3_	8.30	8.32	9.06	9.90	7.72	6.88
Lys	1.80	8.97	10.08	11.15	9.66	9.83
Met	1.50	2.68	2.70	2.78	2.66	2.68
Thr	0.10	3.60	4.01	4.37	3.83	3.91
Trp		0.93	1.10	1.23	1.00	1.00
Val		3.58	4.00	4.34	3.82	3.93
Arg		6.05	7.03	8.03	6.68	6.88
Cys		2.15	2.22	2.38	2.12	2.12
Gly		5.86	6.43	6.53	7.00	6.80
His		0.96	1.16	1.41	1.12	1.21
Ile		3.26	3.7	4.14	3.65	3.83
Leu		2.40	2.40	2.64	2.98	3.72
Phe		2.77	3.14	3.53	3.27	3.59
Ser		3.40	2.80	3.00	2.00	2.00
Titanium dioxide	3.0	3.0	3.0	3.0	3.0	3.0
NaHCO_3_	2.00	2.00	2.00	2.00	2.00	2.00
Salt	3.50	3.50	3.50	3.00	3.50	3.50
Vitamin premix ^1^	5.00	5.00	5.00	5.00	5.00	5.00
Trace minerals premix ^2^	5.00	5.00	5.00	5.00	5.00	5.00
Phytase	0.10	0.10	0.10	0.10	0.10	0.10
Total	1000	1000	1000	1000	1000	1000
Calculated composition and digestible AA						
Protein	185	141	141	141	141	141
ME, kcal/kg	3094	3114	3118	3119	3114	3117
Ca	6.19	6.20	6.20	6.20	6.20	6.20
P	4.87	4.16	4.14	3.97	4.36	4.45
Available P ^3^	2.51	2.50	2.50	2.50	2.50	2.50
Arg	11.9	11.0	11.0	11.0	11.0	11.0
His	4.94	3.51	3.50	3.50	3.51	3.50
Ile	7.74	6.90	6.90	6.90	6.90	6.90
Leu	16.3	12.0	12.0	12.0	12.0	12.0
Lys	11.0	11.0	11.0	11.0	11.0	11.0
Met	4.45	4.41	4.41	4.40	4.40	4.40
Cys	3.08	3.81	3.80	3.80	3.80	3.80
Phe	8.96	7.20	7.20	7.20	7.20	7.20
Thr	6.99	7.00	7.05	7.00	7.00	7.00
Trp	2.06	1.81	1.82	1.80	1.80	1.81
Val	8.69	8.00	8.07	8.00	8.01	8.00
TSAA	7.52	8.22	8.20	8.20	8.20	8.20
Phe+Tyr	15.6	10.6	10.4	10.1	10.3	10.1
Gly-equivalent	9.67	12.21	11.96	12.10	12.14	12.17

^1^ Vitamin A, 3,527,360 IU/kg; vitamin D3, 1,399,921 ICU/kg; vitamin E, 19,400 IU/kg; vitamin B12, 8.8 mg/kg; menadione, 1102 mg/kg; riboflavin, 3527 mg/kg; d-pantothenic acid, 5467 mg/kg; thiamine, 970 mg/kg; niacin, 20,282 mg/kg; vitamin B6. 1455 mg/kg; folic acid, 573 mg/kg; biotin, 79 mg/kg. µg. ^2^ Calcium, 3.20–4.20%; manganese 13.40%; Zinc, 10.70%; magnesium, 2.68%; iron, 2.63%; copper, 40,000 ppm; iodine, 1000 ppm; selenium, 400 ppm. ^3^ Available P level included the matrix for the phytase. PC—adequate protein, NC—low protein CP—crude protein; CM—canola meal (included at 50 or 100 g/kg); SBM—soybean meal; cDDGS—Corn distillers dried grain (included at 50 or 125 g/kg); Gly-equivalent = Gly + (Ser × 0.714).

**Table 4:** Feedstuffs and chemical compositions (g/kg) of finisher phase diets (d 28 to 42) fed to broilers receiving diets containing soybean with reduction in crude protein or low-protein diets with serial inclusion levels of canola meal or corn–DDGS supplemented with crystalline amino acids.

Items	PC	NC	cDDGS50	cDDGS125	CM50	CM100
Corn	638	731	715	674	721	697
Corn–DDGS			50	125		
Canola meal					50	100
SBM	238	98	64	15	55	16
Soybean oil	42.0	37	34	32.8	37	39
Cellulose		38	40	47	40	45
Corn starch	50.0	36	33	37	34	38.8
Dicalcium phosphate	7.20	8.6	7.7	6.6	8.35	8.2
CaCO_3_	8.10	8.15	8.85	9.9	7.6	7
L-Lys	1.20	6.3	7.26	8.7	6.9	7.4
DL-Met	1.30	2.05	2.05	2.12	2.02	2
L-Thr	0.70	2.38	2.7	3.16	2.52	2.68
L-Trp		0.65	0.78	1	0.74	0.77
L-Val		2.2	2.5	2.94	2.35	2.5
L-Arg		3.95	4.78	6.02	4.48	4.94
L-Cys		1.58	1.68	1.8	1.56	1.53
L-Gly		4.7	4	4.4	3.9	3.8
L-His		0.6	0.73	1.03	0.7	0.83
L-Ile		2.12	2.5	3.1	2.5	2.77
L-Leu		1.3	1.3	1.5	1.85	2.2
L-Phe		2.06	2.4	2.85	2.5	2.9
L-Ser	1	1	2	2	2	2
NaHCO_3_	2	2	2	2	2	2
NaCl	3	3	3	3	3	3
Vitamin premix ^1^	2.5	2.5	2.5	2.5	2.5	2.5
Trace minerals premix ^2^	5	5	5	5	5	5
Phytase	0.1	0.1	0.1	0.1	0.1	0.1
Total	1000	1000	1000	1000	1000	1000
Calculated composition						
Protein	172	140	140	140	140	140
ME, kcal/kg	3159	3154	3151	3155	3153	3151
Ca	5.82	5.81	5.78	5.80	5.81	5.82
Available P ^3^	2.30	2.32	2.30	2.30	2.30	2.30
Arg	10.9	10.2	10.2	10.2	10.2	10.2
His	4.56	3.55	3.50	3.51	3.52	3.53
Ile	7.10	6.41	6.40	6.42	6.44	6.41
Leu	15.2	12.2	12.2	12.3	12.3	12.1
Lys	9.67	9.73	9.72	9.71	9.72	9.70
Met	4.04	4.02	4.00	4.01	4.01	4.01
Cys	2.86	3.60	3.62	3.61	3.61	3.61
Phe	8.24	7.24	7.26	7.20	7.22	7.21
Thr	7.04	6.35	6.35	6.31	6.30	6.30
Trp	1.88	1.71	1.70	1.71	1.73	1.71
Val	8.02	7.36	7.35	7.30	7.31	7.30
Met+Cys	6.90	7.62	7.62	7.62	7.62	7.62
Phe+Tyr	14.4	11.2	11.0	10.6	10.9	10.6
Gly-equivalent	9.52	10.16	9.88	9.87	9.92	9.75

^1^ Vitamin A, 3,527,360 IU/kg; vitamin D3, 1,399,921 ICU/kg; vitamin E, 19,400 IU/kg; vitamin B12, 8.8 mg/kg; menadione, 1102 mg/kg; riboflavin, 3527 mg/kg; d-pantothenic acid, 5467 mg/kg; thiamine, 970 mg/kg; niacin, 20,282 mg/kg; vitamin B6. 1455 mg/kg; folic acid, 573 mg/kg; biotin, 79 mg/kg. µg. ^2^ Calcium, 3.20–4.20%; manganese 13.40%; Zinc, 10.70%; magnesium, 2.68%; iron, 2.63%; copper, 40,000 ppm; iodine, 1000 ppm; selenium, 400 ppm.^3^ Available P level included the matrix for the phytase. Gly-equivalent = Gly + (Ser × 0.714) PC—adequate protein, NC—low protein. CP—crude protein; CM—canola meal (included at 50 or 100 g/kg); SBM—soybean meal; cDDGS—Corn distillers dried grain (included at 50 or 125 g/kg); DCP—Dicalcium phosphate; n is 6 replicate pens with 15 birds per replicate.

**Table 5:** Analyzed crude protein and total amino acids content (g/kg, as fed) of experimental starter grower phase diets (day 10 to 28) fed to broilers receiving diets containing soybean with reduction in crude protein or low-protein diets with serial inclusion levels of canola meal or corn–DDGS supplemented with crystalline amino acids.

Items	Starter	PC	NC	cDDGS50	cDDGS125	CM50	CM100
Dry matter	899	902	900	901	901	892	898
Crude protein	221	194	144	145	149	144	144
Essential AA							
Arg	14.6	12.0	10.9	12.1	11.1	10.5	11.1
His	5.7	4.9	3.5	3.5	3.9	3.5	3.6
Ile	10.3	8.3	6.9	7.3	7.2	6.7	6.9
Leu	18.5	16.0	11.5	11.4	12.5	11.4	11.5
Lys	12.9	11.7	11.4	11.9	13.0	11.5	11.8
Met	4.2	4.4	4.3	4.3	4.2	4.3	4.1
Phe	11.4	9.5	7.6	7.8	8.0	7.3	7.7
Thr	8.5	7.0	6.5	7.0	7.9	6.8	7.3
Trp	2.7	2.0	1.8	1.9	1.8	1.8	2.1
Val	10.6	8.9	7.7	8.1	8.0	7.4	7.6
Nonessential AA							
Ala	10.5	9.3	5.7	5.5	6.1	5.5	5.1
Asp	22.7	18.2	8.0	6.6	5.9	7.1	6.3
Cys	3.5	3.0	3.8	4.1	3.9	3.9	3.8
Glu	39.2	32.6	16.3	14.3	14.5	15.8	14.6
Gly	8.9	7.5	9.2	10.2	9.9	9.8	9.8
Pro	15.4	13.8	8.9	8.9	9.8	8.8	8.2
Ser	9.0	7.2	6.4	5.7	5.6	4.7	4.9
Tyr	7.7	6.5	3.6	3.3	3.5	3.3	3.2

**Table 6:** Analyzed crude protein and total amino acids content (g/kg, as fed) of finisher diets (d 28 to 42) fed to broilers receiving diets containing soybean with reduction in crude protein or low-protein diets with serial inclusion levels of canola meal or corn–DDGS supplemented with crystalline amino acids.

Items	PC	NC	cDDGS50	cDDGS125	CM50	CM100
Dry matter	901	896	901	899	902	900
Crude protein	167	141	142	143	140	140
Essential AA						
Arg	10.7	9.6	9.9	10.6	9.9	10.1
His	4.3	3.5	3.6	3.8	3.4	3.5
Ile	7.3	6.2	6.4	6.8	6.5	6.7
Leu	14.1	11.6	11.9	12.3	11.5	11.2
Lys	10.2	10.0	10.2	10.8	10.1	10.6
Met	3.9	3.9	4.1	3.9	3.8	3.9
Phe	8.5	7.3	7.6	7.9	7.5	7.7
Thr	6.8	6.1	6.4	6.5	6.0	6.1
Trp	2.2	1.8	1.9	2.0	1.9	1.9
Val	7.6	6.7	6.9	7.3	6.8	7.1
Nonessential AA						
Ala	8.3	6.4	6.6	6.7	6.0	5.6
Asp	15.8	9.6	8.6	6.9	7.9	6.9
Cys	2.9	3.4	3.7	3.9	3.6	3.9
Glu	28.7	19.3	18.3	16.4	17.4	16.3
Gly	7.0	8.4	8.0	8.1	7.8	8.0
Pro	11.3	8.9	9.0	9.4	8.7	9.0
Ser	7.7	5.5	6.1	5.6	5.6	5.5
Tyr	5.8	4.1	4.1	3.8	3.6	3.3

**Table 7:** Growth performance of broilers receiving diets containing soybean with reduction in crude protein or low-protein diets with serial inclusion levels of canola meal or corn–DDGS supplemented with crystalline amino acids.

Diets	(Grower Phase) d 10 to 28			(Finisher Phase) d 28 to 42			d42 BW, g
	Weight Gain, g	FI, g	FCR	Weight Gain, g	FI, g	FCR	
Positive control (PC)	893	1346	1.51	1581	2777	1.76	2724
Negative control (NC)	699	1208	1.73	1189	2388	2.01	2154
cDDGS50	633	1140	1.80	1135	2294	2.02	2020
cDDGS125	498	1022	2.21	1037	2107	2.06	1764
CM50	696	1243	1.79	1159	2474	2.15	2123
CM100	619	1169	1.91	1008	2287	2.29	1884
Pooled SEM	34.8	52.0	0.127	59.2	96.8	0.084	82.9
*p* values	<0.001	0.005	0.020	<0.001	0.002	0.005	<0.001
Contrasts							
PC vs. NC	0.021	0.199	<0.001	0.015	0.095	0.008	0.023
cDDGS level—Linear	0.001	0.019	0.099	0.128	0.144	0.688	0.004
cDDGS level—Quadratic	0.388	0.680	0.475	0.787	0.767	0.919	0.516
CM level—Linear	0.095	0.520	0.008	0.024	0.404	0.033	0.036
CM level—Quadratic	0.351	0.314	0.564	0.327	0.199	0.954	0.306

n is 6 replicate pens with 15 birds per replicate. PC—adequate protein, NC—low protein; CP—crude protein; CM—canola meal (included at 50 or 100 g/kg); SBM—soybean meal; cDDGS—Corn distillers dried grain (included at 50 or 125 g/kg); Linear and quadratic contrasts for cDDGS used treatments NC, cDDGS50 and cDDGS 125; Linear and quadratic contrasts for CM used treatments NC, CM50 and CM100.

**Table 8:** Carcass composition at day 42 of broilers receiving diets containing soybean with reduction in crude protein or low-protein diets with serial inclusion levels of canola meal or corn–DDGS supplemented with crystalline amino acids.

Diets	Eviscerated Weight, kg	Abdominal Fat Weight, g	Carcass Yield, %	Fat Yield, %	Breast Yield, %	Wings Yield, %	Thighs Yield, %	Back + Ribs Yield, %
Positive control (PC)	2.05	43.7	75.2	1.61	25.4	10.6	28.3	35.7
Negative control (NC)	1.67	48.4	72.0	2.08	22.4	11.1	30.1	36.4
cDDGS50	1.65	49.3	73.2	2.17	21.7	11.5	29.5	37.3
cDDGS125	1.38	27.8	71.1	1.43	21.9	11.9	29.9	36.2
CM50	1.82	47.8	72.6	1.90	23.1	10.8	29.9	36.2
CM100	1.54	35.9	72.7	1.69	23.46	11.58	29.24	35.73
Pooled SEM	0.046	3.06	0.808	0.121	0.555	0.252	0.524	0.555
*p* values	<0.001	<0.001	0.021	<0.001	0.351	0.003	0.205	0.351
Contrasts								
PC vs. NC	<0.001	0.337	<0.001	0.024	0.464	0.189	0.048	0.464
cDDGS level—Linear	<0.001	<0.001	0.557	0.004	0.787	0.032	0.761	0.787
cDDGS level—Quadratic	0.021	0.017	0.193	0.031	0.113	0.961	0.354	0.113
CM level—Linear	0.043	0.017	0.228	0.034	0.342	0.156	0.160	0.342
CM level—Quadratic	<0.001	0.193	0.627	0.953	0.834	0.080	0.705	0.834

n is 6 replicate pens with 2 birds per replicate. CP—crude protein; CM—canola meal (included at 50 or 100 g/kg); SBM—soybean meal; cDDGS—Corn distillers dried grain (included at 50 or 125 g/kg); PC—adequate protein, NC—low protein; Linear and quadratic contrasts for cDDGS used treatments NC, cDDGS50 and cDDGS 125; Linear and quadratic contrasts for CM used treatments NC, CM50 and CM100.

**Table 9:** Whole body composition (d 21) of broilers receiving diets containing soybean with reduction in crude protein or low-protein diets with serial inclusion levels of canola meal or corn–DDGS supplemented with crystalline amino acids.

Diets	BMD, g/cm^3^	BMC, g	Bone Area, cm^2^	Fat wt., kg	Lean wt., kg	Fat, %	Lean Muscle, %
Positive control (PC)	0.114	6.67	56.2	0.07	0.49	10.8	89.2
Negative control (NC)	0.126	8.15	63.5	0.10	0.54	15.7	84.4
cDDGS50	0.113	6.08	52.8	0.07	0.44	13.8	86.2
cDDGS125	0.108	5.57	50.7	0.06	0.42	11.6	88.2
CM50	0.114	6.88	59.2	0.08	0.49	13.6	86.5
CM100	0.122	7.48	60.3	0.09	0.51	14.3	85.7
Pooled SEM	0.006	1.025	6.13	0.034	0.111	1.32	1.319
*p* values	0.297	0.542	0.687	0.447	0.574	0.150	0.156
Contrasts							
PC vs. NC	0.201	0.378	0.454	0.164	0.431	0.040	0.042
cDDGS level—Linear	0.021	0.020	0.034	0.014	0.013	0.020	0.023
cDDGS level—Quadratic	0.497	0.362	0.369	0.587	0.349	0.980	0.966
CM level—Linear	0.649	0.690	0.728	0.494	0.673	0.420	0.430
CM level—Quadratic	0.273	0.522	0.727	0.559	0.570	0.320	0.319

n is 6 replicate pens with 15 birds per replicate. BMD—bone mineral density; BMC—bone mineral content; CP—crude protein; CM—canola meal (included at 50 or 100 g/kg); SBM—soybean meal; cDDGS—Corn distillers dried grain (included at 50 or 125 g/kg); PC—adequate protein, NC—low protein; Linear and quadratic contrasts for cDDGS used treatments NC, cDDGS50 and cDDGS 125; Linear and quadratic contrasts for CM used treatments NC, CM50 and CM100.

**Table 10:** Whole body composition (d 42) of broilers receiving diets containing soybean with reduction in crude protein or low-protein diets with serial inclusion levels of canola meal or corn–DDGS supplemented with crystalline amino acids.

Diets	BMD, g/cm^3^	BMC, g	Bone Area, cm^2^	Fat wt., kg	Lean wt., kg	Fat, %	Lean Muscle, %
Positive control (PC)	0.200	39.5	198	0.53	1.97	21.2	78.8
Negative control (NC)	0.170	28.2	163	0.39	1.45	21.1	78.9
NC + cDDGS50	0.180	28.7	161	0.39	1.52	20.4	79.7
NC + cDDGS125	0.170	26.5	159	0.35	1.43	19.3	80.8
NC + CM50	0.170	27.1	163	0.36	1.46	19.6	80.4
NC + CM100	0.160	24.7	151	0.31	1.29	19.4	80.6
Pooled SEM	0.007	2.24	10.4	0.081	0.216	0.889	0.891
*p*-value	0.013	0.002	0.044	0.010	0.001	0.481	0.475
Contrasts							
PC vs. NC	0.009	0.030	0.087	0.086	0.030	0.919	0.915
cDDGS level—Linear	0.648	0.622	0.764	0.500	0.892	0.251	0.248
cDDGS level—Quadratic	0.404	0.650	0.995	0.664	0.576	0.885	0.887
CM level—Linear	0.317	0.251	0.375	0.078	0.192	0.217	0.217
CM level—Quadratic	0.815	0.825	0.624	0.800	0.381	0.548	0.554

n is 6 replicate pens with 15 birds per replicate. BMD—bone mineral density; BMC—bone mineral content; CP—crude protein; CM—canola meal (included at 50 or 100 g/kg); SBM—soybean meal; cDDGS—Corn distillers dried grain (included at 50 or 125 g/kg); PC—adequate protein, NC—low protein; Linear and quadratic contrasts for cDDGS used treatments NC, cDDGS50 and cDDGS 125; Linear and quadratic contrasts for CM used treatments NC, CM50 and CM100.

**Table 11:** Litter ammonia and N measured at d 42 of broilers receiving diets containing soybean with reduction in crude protein or low-protein diets with serial inclusion levels of canola meal or corn–DDGS supplemented with crystalline amino acids.

Diets	Litter NH_3_, ppm	Litter N, %	Litter N/kg Body Weight
Positive control (PC)	50.5	2.24	24.7
Negative control (NC)	10.4	1.83	20.2
cDDGS50	13.5	1.82	20.7
cDDGS125	15.8	1.80	21.4
CM50	11.4	1.85	20.8
CM100	8.98	1.89	21.6
Pooled SEM	6.93	0.232	2.05
*p* values	<0.001	0.923	0.682
Contrasts			
PC vs. NC	0.041	0.532	0.240
cDDGS level—Linear	0.399	0.727	0.607
cDDGS level—Quadratic	0.944	0.631	0.947
CM level—Linear	0.461	0.386	0.554
CM level—Quadratic	0.303	0.540	0.939

n is 6 replicate pens with 15 birds per replicate. CP—crude protein; CM—canola meal (included at 50 or 100 g/kg); SBM—soybean meal; cDDGS—Corn distillers dried grain (included at 50 or 125 g/kg); PC—adequate protein; NC—low protein; Linear and quadratic contrasts for cDDGS used treatments NC, cDDGS50 and cDDGS 125; Linear and quadratic contrasts for CM used treatments NC, CM50 and CM100.

## Data Availability

The data presented in this study are available in supplementary material.

## Supplementary Materials

The following supporting information can be downloaded at: https://www.mdpi.com/article/10.3390/ani12192662/s1, Table S1: Data analysis.
